# Rubber plantations and drug resistant malaria: a cross-sectional survey in Cambodia

**DOI:** 10.1186/s12936-019-3000-y

**Published:** 2019-11-27

**Authors:** Rebecca Thomson, Phok Sochea, Mak Sarath, Amanda MacDonald, Abigail Pratt, Steve Poyer, Henrietta Allen, Sok Kunthy, Sok Chamroeun, Kim Daro, Sourn Samean, Nou Panharith, Sok Ra, Chan Sovottha, Gary Mundy, Shunmay Yeung

**Affiliations:** 1 Department of Global Health and Development, Faculty of Public Health and Policy, London School of Hygiene and Tropical Medicine, London, UK; 2 Population Services Khmer, Phnom Penh, Cambodia; 3 Population Services International, Washington, DC USA; 4 Partners for Development-LSHTM Partnership, Phnom Penh, Cambodia; 5 Helen Keller International, New York, USA; 6 Clinical Research Department, Faculty of Infectious and Tropical Disease, London School of Hygiene and Tropical Medicine, London, UK

**Keywords:** Forest, Malaria, Artemisinin resistance, Plantations, Asia, Cambodia

## Abstract

**Background:**

The ongoing spread of artemisinin resistant *Plasmodium falciparum* malaria is a major threat to global health. In response, countries in the Greater Mekong Sub-region, including Cambodia, have declared ambitious goals to eliminate malaria. Major challenges include the lack of information on the at-risk population-individuals who live or work in or near the forest where the malaria vectors are found, including plantation workers. This study aimed to address this knowledge gap through a cross-sectional survey conducted in rubber plantations in Cambodia in 2014.

**Methods:**

The survey was conducted in two rounds in four provinces and included a malaria prevalence survey, analysis for the K13 genetic mutation, and a comprehensive behavioural questionnaire. Forty plantations were included in each round, and 4201 interviews were conducted. An additional 701 blood samples were collected from family members of plantation workers.

**Results:**

Overall malaria prevalence was relatively low with adjusted PCR prevalence rate of 0.6% for *P. falciparum* and 0.3% for *Plasmodium vivax*, and was very heterogenous between plantations. There was little difference in risk between permanent residents and temporary workers, and between the two rounds. The main risk factors for *P. falciparum* infection were smaller plantations, age under 30 years, lack of self-reported use of a treated net and recent travel, especially to the Northeastern provinces. Proximity of plantations to the forest was also a risk factor for malaria in round one, while male gender was also a risk factor for malaria by either species.

**Conclusions:**

With Cambodia’s *P. falciparum* elimination target on the horizon, identifying every single malaria case will become increasingly important. Plantations workers are relatively accessible compared to some other at-risk groups and will likely remain a high priority. Ongoing surveillance and adaptive strategies will be critical if malaria elimination is to be achieved in this setting.

## Background

Malaria control efforts in Cambodia have led to a substantial decrease in cases over the past 15 years [1]. However, this progress is threatened by artemisinin-resistant *Plasmodium falciparum,* which was first detected along the Cambodia–Thailand border in the late 2000s [2] and has since been identified in Thailand, Myanmar, Vietnam and Laos [3–5]. Should artemisinin-resistant malaria spread from this region to Africa, as did resistance to chloroquine and sulfadoxine–pyrimethamine [6], much of the progress made against malaria in the last decade could be reversed [4, 7], putting millions of lives at risk [8].

In response, the World Health Organization and other partners have launched several initiatives to tackle artemisinin resistance [9], and in 2014 the Cambodian government declared a goal for malaria elimination by 2025 [10]. One of the central challenges to malaria control and elimination in this region is that vectors that transmit malaria live in the forest. This means that the most at-risk population are individuals who live and work in remote forested areas and are often hard to reach [11–13]. These at-risk populations include local residents as well as mobile and migrant populations (MMP).

Approximately 27% of the population, or about 3.5 million people, in Cambodia are estimated to be migrant, defined as ‘a person who has moved to their enumeration area from another village or another country, which was the person’s last residence’ [14, 15]. The MMP who go into forested areas for work include security personnel, loggers, construction workers and seasonal plantation workers. Those arriving in forested areas from non-forested areas are often biologically more susceptible to malaria than local communities who have developed partial immunity [16, 17] and have also been shown to have a higher risk of infection [18–20] due to inconsistent bed net use and poor access to health care [21, 22]. The MMP are, therefore, a key affected population for malaria control programmes and a vital group to reach if elimination aspirations are to be attained [9].

Rubber plantation workers account for a significant proportion of MMPs working in forested areas in Cambodia [23]. Access to seasonal plantation workers may be easier than to other MMPs such as security personnel and loggers. Studies in neighbouring countries have shown that plantation workers have an increased exposure to the *Anopheles* vectors and that working conditions are often poor. Rubber tapping is carried out at night, exposing workers to nocturnally active exophilic mosquito vectors [24] and limiting the effectiveness of insecticide-treated nets (ITNs) [25]. They are thus at higher risk of malaria than other people living in the area [20, 26].

Rubber plantations have been shown to create situations of emergence and re-emergence of malaria in the region, with transient workers being an integral part of the malaria transmission cycle [27]. Biological, social and economic factors, such as behaviour, mobility patterns and access to health care, along with plantation characteristics all play a role in the level of vulnerability to malaria infection. This web of factors, along with the transient nature of many of the workers, makes it challenging to determine the most effective approach to target these populations. Common malaria control interventions such as case management through village malaria workers (VMWs) and village based distribution of insecticide-treated nets mainly focus on stable, village-based populations and may miss at-risk rubber plantation workers.

One approach that has been tried in Cambodia is a Plantation Malaria Worker (PMW) model. This is based on the village malaria worker (VMW) programme which has been implemented by the Cambodian National Centre for Parasitology, Entomology and Malaria Control (CNM) since 2001 [28], with an aim of improving free access to malaria diagnosis and treatment in remote areas. A number of CNM partners have since launched similar programmes to complement the existing VMW network including Population Services Khmer (PSI/PSK) who launched a PMW programme in 2013. At each of the participating plantations one to three workers are trained in malaria case management and stocked with malaria rapid diagnostic test kits (RDTs) and quality-assured artemisinin-based combination therapy free of charge. The PMW is required to complete a Patient Register Form to track each suspected and confirmed case and also supports the distribution of ITNs to plantation workers.

The VMW and PMW programmes have proven successful in providing access to quality assured diagnosis and treatment of symptomatic malaria. However, in the context of malaria elimination, it is felt that interventions such as mass drug administration or active screening and treatment may be needed in order to substantially reduce the reservoir of infection in asymptomatic malaria carriers [28, 29]. There is, however, a lack of relevant information for the design of such interventions and how to target the most at-risk populations, including basic information such as who exactly is at risk, their work and travel patterns, and their knowledge and behaviours in relation to malaria risk, prevention and treatment.

In order to address this knowledge gap, an epidemiological survey was conducted in 42 plantations involved in PSK’s PMW programme. The study aimed to document the prevalence of malaria and artemisinin resistance on plantations and to examine the potential risk factors for infection in order to inform the design of feasible and appropriate interventions to targeting plantation workers. A secondary objective was to describe the prevalence of malaria in non-plantation-working household members of plantation workers in order to inform how actively they should be included in future interventions.

## Methods

### Study design and population

This was a cross-sectional survey of malaria prevalence, behaviour and knowledge in plantation workers. The survey was carried out in two rounds in recognition of the seasonal nature of plantation work and malaria transmission. Round 1 was conducted from June 1 to July 3, 2014 at the beginning of the wet season and Round 2 was conducted from October 1 to October 27, 2014, in the middle of the wet season.

**Fig. 1:**
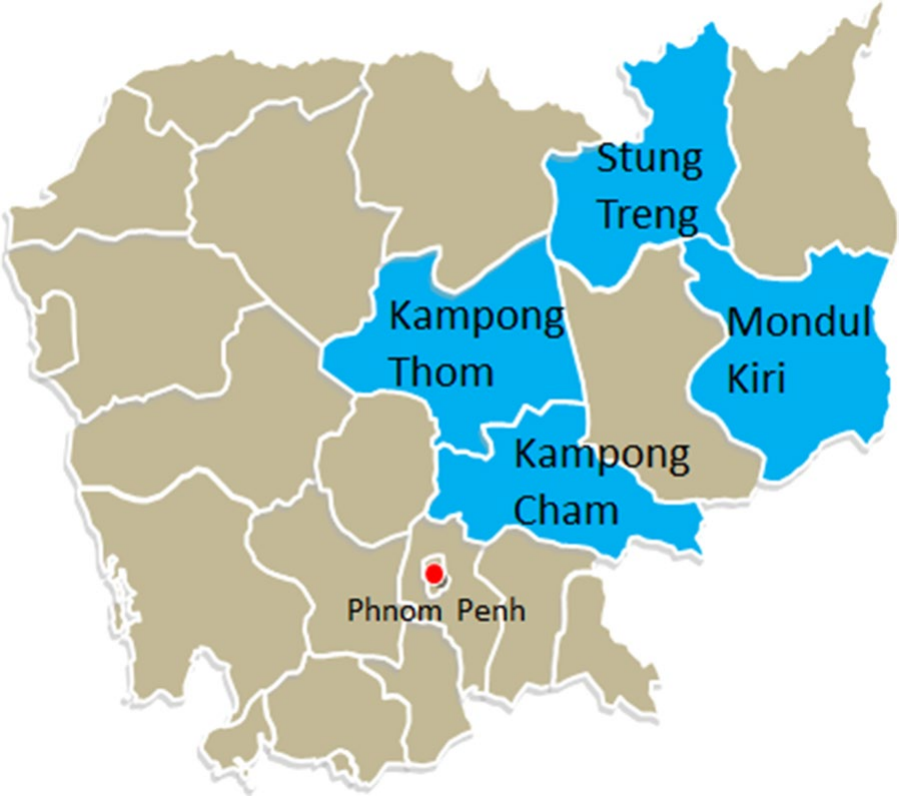
Map of Cambodia showing which provinces data collection was conducted in

Forty plantations in four provinces were visited in each round (Fig. 1). In the 2nd round access was denied to two plantations which had been included in the 1st round and were replaced by plantations with similar characteristics. Therefore, in total 42 different plantations were visited, with 38 plantations being visited in both rounds, and four plantations only visited once.

All of the plantations involved in PSK’s PMW programme were eligible for inclusion. At the initiation of the study there were 42 plantations of which 40 agreed to participate. The PMW programme was expanding during the time of the survey, and therefore two plantations which had recently joined the PSK’s PMW programme were included as replacements during the 2nd round. The original sample size was based on comparing malaria in permanent *versus* temporary workers. It was estimated that the prevalence in permanent workers would be around 1.0% and that there would be a minimum difference of 2.1% with temporary workers (Alpha error level of 95%; Beta error level of 80%; Design effect of 1.5 and dropout rate of 5%). A minimum sample size of 1273 was required for each study strata, or 32 of each type of worker from each of the 40 plantations. In order to estimate the prevalence of malaria among household members of plantation workers, a sample of 560 households members (14 per plantation) were required, to provide estimates of malaria prevalence accurate to within ± 1.5% points, assuming a prevalence point estimate of 2%. Family members had to be 5 years or above and not working in the plantation.

**Fig. 2:**
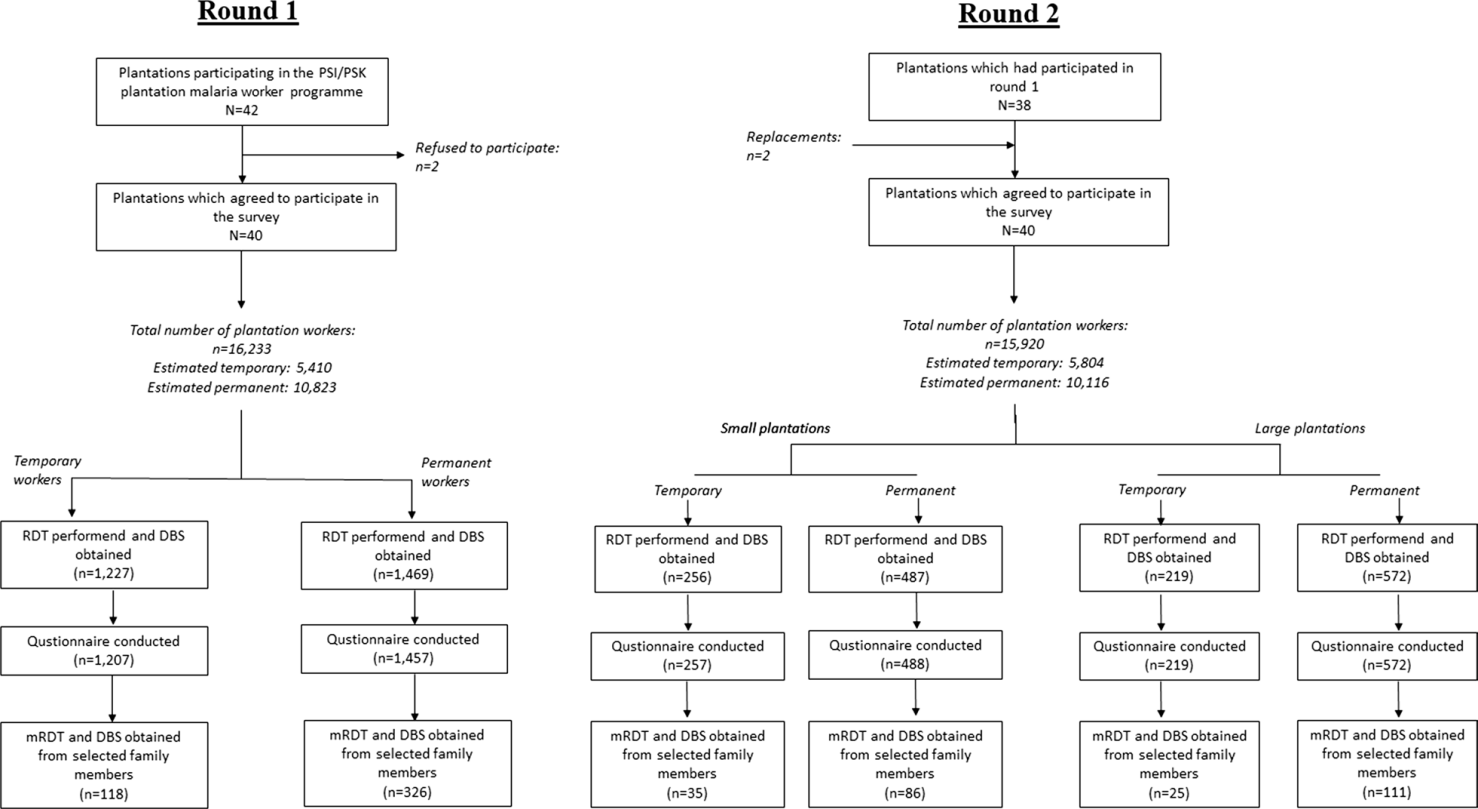
Flow chart to show selection of plantations and participants. *DBS* dried blood spot, *RDT* rapid diagnostic test

Analysis of data from the first round showed that there was no statistical difference in malaria prevalence between permanent and temporary workers. The only statistically significant factor associated with malaria prevalence was the size of the plantation with smaller plantations (less than 6200 ha) being associated with a higher prevalence of malaria. For the second round, the sampling was adjusted to ensure that a comparison could also be made between smaller and larger plantations, as well as between permanent and temporary workers. This resulted in a required sample size of 1504, based on the survey including 25 small plantations and 15 big plantations, alpha error level of 95%, beta error level of 80% and an assumed dropout rate of 10%. From small plantations, the target sample size of 32 workers were sampled (15 temporary and 17 permanent) and on large plantations 48 workers (15 temporary and 33 permanent workers) (Fig. 2).

### Questionnaire design and piloting

Data were collected using a paper-based structured questionnaire which collected information on socio-demographics, work patterns, knowledge of malaria and malaria prevention methods. Additional modules were asked of those who reported fever in the previous 14 days (“treatment seeking module”); had travelled to another commune in the previous 6 months (“mobility module”); or who had been into the forest in the previous month (“forest module”). The questionnaire was designed in English, translated into Khmer, back translated and piloted several times before being used.

### Survey team

Prior to field the survey implementation, a team from PSK visited each plantation to inform the plantation manager or owner about the upcoming study and ask for approval to conduct the survey.

The survey was conducted by four data collection teams consisting of eight or nine members including two supervisors, and four ‘advance teams’ consisting of four of five members. The survey team received 1 week of training and participated in a fieldwork practice.

### Census and enrolment

The advance teams visited plantations a few days before the arrival of the survey team to select, consent and enrol participants. Where the plantation manager had an up-to-date list of both permanent and temporary workers, this was used. Where no such list existed, a full census was undertaken with the help of the plantation managers who listed the workers and classified them as temporary (< 6 months) or permanent (> 6 months) according to their time working on site. The required sample of workers was then randomly selected from each group using simple random selection. On larger plantations this listing was facilitated with input from work team supervisors in the plantation, who have a closer day-to-day relationship with workers in the team than the plantation managers. The selected workers were visited and provided with verbal and written information about the survey and asked to give their written (or thumbprint) consent if they agreed to participate and were asked further information in order to confirm correct classification of their residency status. Participants were then informed of the time, date and location at which the survey would take place on the plantation.

In five plantations access to the workers by the survey team was restricted by the plantation management because of the sensitive nature of some of the plantation’s activities. This is explained further in “Discussion”. In these cases the participants were selected by the management and the randomized selection process could not be followed.

### Data collection

On the day of the survey, participants arriving at the survey site were registered and cross-checked against the list drawn up by the advance team. Participants then had their temperature taken and finger prick blood sample taken for malaria RDT and one dried blood spot collected on filter paper for later analysis by polymerase chain reaction (PCR).

All participants testing positive for malaria by RDT were offered immediate treatment with dihydroartemisinin–piperaquine, the national first-line treatment at the time of the study. The structured questionnaire was then administered to each worker by a data collector. The first 14 accompanying adult family members (one per worker) were provided with information about the survey and, if they consented, had blood taken for malaria, but did not undertake the survey questionnaire.

### Laboratory analysis

All blood samples for PCR analysis were transported to Institut Pasteur du Cambodge in Phnom Penh twice per week and analysed for confirmation of *Plasmodium*, species identification and presence of resistance marker mutations using methods previously described by Canier et al. [30]. All *P. falciparum* positive samples were then processed to look for K13 mutations [31]. Discordant samples were rerun for quality control.

### Data analysis

**Fig. 3:**
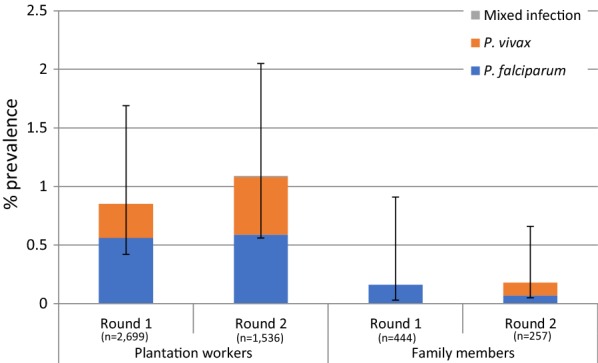
Malaria prevalence by PCR in Round 1 (June 2014) and Round 2 (October 2014) by *Plasmodium* species

Data were double entered using EpiData with in-built checks for consistency and range values. Each survey dataset was validated and all inconsistencies were checked against the original questionnaires and amended. Data were linked to the blood results using a unique identifier code and analysed in Stata version 12 (Stat Corp, College Station, Texas). Stata survey design commands were used to account for clustering, stratification and weighting. Each participant was given a weight based on their probability of being selected into the study, calculated separately for each classification of residence status and the size of that worker population on the plantation. Differences in malaria prevalence by risk factor were calculated using the design based F-test. Results presented on plantation level characteristics (Table 2) use raw data, while results presented from Table 3 onwards uses weighted data. With the exception of Fig. 3, where the PCR prevalence among family members is presented, all results presented include data from plantation workers only, exclusive of family members.

For the multivariate model of risk for malaria, a priori variables included the round, plantation size and proximity to the natural forest and individual worker characteristics, i.e. age, gender, residence status, recent forest exposure, recent travel history and reported habitual insecticide-treated net usage. The variable for having slept under a treated net the previous night was not included due to colinearity with the variable for habitual insecticide-treated net usage.

Data on plantation age and size was obtained from the plantation management. Age of plantation was based on the year the business started. For plantation size, analysis was performed using the square root of the plantation area to provide an indication of the average distance of workers inside the plantation to the plantation’s edge. This was then divided by 10 so the effect of the change in size could be assessed more clearly. Forest “proximity” was defined as the proportion of the land within 5 km of the plantation boundary that consisted of dense or mixed forest and was estimated from publicly available map data [32]. Map data were not available for five plantations.

The classification of residency status for data analysis was based on the workers own responses, rather than the original classification by the plantation manager and advance team. Workers were classified as “temporary” if they reported having lived in the commune where the plantation was located for less than 6 months or if they reported having worked on another plantation in the previous 6 months. Workers were classified as “permanent” if they had lived in their current living commune and had not worked in another plantation in the last 6 months.

### Ethical approval

Ethical approval was obtained from the National Ethics Committee for Health Research of Cambodia; Population Services International’s Institutional Review Board; and the London School of Tropical Medicine and Hygiene’s ethics committee (Ref: 7607).

## Results

### Sample description

A total of 4201 workers were interviewed: 2664 in the first round and 1537 in the second round (Table 1). An additional 35 workers in round one had blood taken without interview due to time constraints. Four hundred and four family members had blood taken in round one and 257 in round two.

**Table 1:** Description of sample

Round 1 (June 2014)				
Province	Number of plantations visited	Number of plantation workers interviewed		
		Temporary^a^	Permanent	Total
Kampong Cham	13	273	613	886
Kampong Thom	11	403	404	807
Mondul Kiri	9	308	188	496
Stung Treng	7	223	252	475
Total	40	1207	1457	2664
		Number of blood samples taken		
Plantation workers		1227	1469	2699^b^
Family members		118	326	444
Total		1345	1795	3143^b^

Round 2 (October 2014)				
Province	Number of plantations visited	Number of plantation workers interviewed		
		Temporary^a^	Permanent	Total^c^
Kampong Cham	13	74	387	461
Kampong Thom	11	155	278	433
Mondul Kiri	8	134	190	325
Stung Treng	8	113	205	318
Total	40	476	1060	1537
		Number of blood sample taken		
Plantation workers		475	1057	1534^d^
Family members		60	197	257
Total		535	1254	1793^d^

^a^Based on classification from questionnaire data

^b^35 plantation workers had blood samples taken but were not interviewed due to limited time availability. These workers could not be classified as temporary or permanent

^c^Residence status could not be determined for one worker interviewed in round two as data were missing from the required questions to categorize them

^d^Two plantation workers did not have blood taken due to lack of consent, while one could not be categorized as temporary or permanent

### Plantation characteristics

Rubber was the predominant crop, with 22/42 plantations growing only rubber, 13/42 growing a mixture of rubber and cassava and seven growing rubber plus another crop including sugarcane and watermelon. The median age of the plantations was 7 years (range 2–17 years). The size of the selected plantations ranged from 80 to 40,000 hectares, with a median of 5214 hectares (Table 2). The percentage of the area surrounding each plantation that was forested ranged from 26 to 89%.

**Table 2:** Characteristics of plantations

	Round 1 (June 2014) Median (range)	Round 2 (October 2014) Median (range)
Size in hectares	5214 (80–40,000) ha	5214 (150–40,000) ha
Age in years	7 (2–17) years	7 (2–17) years
% of area surrounding plantation covered by forest	63% (26–89%)	63% (26–89%)
Number of workers	250 (19–3339)	209 (19–3339)
% temporary based on questionnaire data	45% (15–100%)	31% (8–78%)

### Worker demographics, accommodation and work type

The number of workers reported on site varied between 19 and 3339. Based on questionnaire data, 1207 (median 45%, range 15–100%) workers were classified as temporary and 1457 as permanent in round one, and 476 (median 31%, range 8–78%) and 1060, respectively, in round two. Residence status could not be determined for one worker in round two due to missing information. Overall just over half the plantation workers were male, and half were aged between 15 and 30 years (Table 3). The level of education was generally low with over two thirds of workers reporting that they had not completed primary school. The median household size was four people. Almost all workers (94%) self-identified as Khmer, with the remainder identifying as Cham (5%) or Vietnamese. Just under half of the workers lived in a house while about 46% lived in a barrack, and the remaining 7% lived in tents or temporary structures. The main types of work during the day included tapping rubber (45%), and planting or caring for young plants (41%). Just over half (55%) of the workers reported working at night, during which time rubber tapping was the main activity (40%). There was no significant difference between rounds for any of these factors, nor between temporary and permanent workers, except that temporary workers were more likely than permanent workers to live in a tent or a temporary structure (15% and 3%), and less likely to live in a house (40% and 51%) (p = 0.012) (results not shown).

**Table 3:** Characteristics of plantation workers

	Round 1 (June 2014)		Round 2 (October 2014)		Overall	
	N	% (95% CIs)	N	% (95% CIs)	N	% (95% CIs)
Gender						
Male	1493	56.8 (53.0–60.4)	938	58.7 (55.3–62.0)	2431	57.7 (54.8–60.5)
Female	1171	43.3 (39.6–47.0)	599	41.3 (38.0–44.8)	1770	42.3 (39.5–45.2)
Age group						
15–30	1405	49.4 (41.1–57.6)	804	51.8 (46.1–57.4)	2209	50.5 (44.0–57.0)
31+	1259	50.7 (42.4–58.9)	732	48.2 (42.6–53.9)	1991	49.5 (43.0–56.0)
Education						
No or some primary	1839	71.1 (67.6–74.5)	1088	68.9 (64.6–72.9)	2927	70.0 (67.6–72.4)
Some Secondary	613	21.6 (18.8–24.8)	347	25.0 (21.4–29.0)	960	23.3 (21.4–25.3)
Completed secondary or higher	211	7.2 (5.5–9.4)	102	6.1 (4.8–7.6)	313	6.7 (5.7–7.8)
Residence status based on questionnaire data						
Temporary	1207	34.9 (26.2–44.8)	476	21.6 (13.4–33.1)	1683	28.4 (20.2–38.4)*
Permanent	1457	65.1 (55.2–73.8)	1060	78.4 (66.9–86.6)	2517	71.6 (61.6–79.8)
Type of house						
House	1438	44.4 (31.7–57.9)	858	51.3 (36.3–66.1)	2296	47.8 (35.0–60.9)
Barrack	990	50.9 (36.9–64.7)	498	40.4 (24.3–58.8)	1488	45.8 (31.5–60.7)
Tent or temporary structure	235	4.7 (2.4–8.9)	180	8.3 (3.6–17.9)	415	6.5 (3.4–12.1)
Reported habitual use of insecticide treated net as a malaria prevention method at night						
Yes	795	33.1 (28.9–37.7)	908	56.1 (49.7–62.2)	1703	44.3 (39.6–49.2)*
No	1869	66.9 (62.3–71.2)	629	43.9 (37.8–50.3)	2498	55.7 (50.8–60.4)
Use of treated net the previous night						
Yes	927	35.7 (32.2–39.3)	972	61.0 (56.5–65.3)	1899	48.0 (44.7–51.4)*
No	1736	64.3 (60.7–67.8)	565	39.0 (34.7–43.5)	2301	51.9 (48.6–55.3)
Any Forest exposure in the last 1 month						
Yes	747	22.4 (12.8–36.2)	370	18.5 (15.2–22.2)	1117	20.5 (14.2–28.6)
No	1917	77.6 (63.8–87.2)	1167	81.6 (77.8–84.8)	3084	79.5 (71.4–85.8)
Overnight forest exposure in last 1 month						
Yes	25	0.8 (0.3–1.7)	74	2.6 (1.5–4.6)	99	1.7 (1.0–2.8)*
No	2639	99.2(98.2–99.7)	1463	97.4(95.4–98.6)	4102	98.3 (97.2–99.0)
Main daytime work						
Tapping rubber	743	44.6 (30.5–59.7)	372	45.7 (30.1–62.3)	1115	45.2 (31.2–60.0)
Planting/caring for young plants	1142	38.4 (25.4–53.3)	941	44.3 (27.9–62.0)	2083	41.3 (27.7–56.4)
Clearing forest	292	4.8 (2.8–8.2)	12	0.3 (0.1–0.7)	304	2.6 (1.5–4.4)
Other/does not work	487	12.5 (9.1–16.1)	212	9.7 (7.4–12.6)	699	11.0 (8.9––13.6)
Main nighttime work						
Tapping rubber	657	42.7 (28.3–58.8)	292	37.1 (22.7–54.3)	949	40.0 (26.5–55.2)
Does not work	1851	51.5 (34.7–67.9)	1181	57.8 (41.1–72.8)	3032	54.6 (38.9–69.4)
Other	156	5.8 (3.7–9.0)	63	5.1 (3.0–8.5)	219	5.4 (3.8–7.7)
Travelled outside of the commune in the previous 1 month						
Yes	211	9.4 (6.6–13.3)	583	34.8 (29.5–40.5)	794	21.8 (18.7–25.3)*
No	2453	90.6 (86.7–93.4)	954	65.3 (59.6–70.5)	3407	78.2 (74.7–81.3)
Travel to another country in the previous 1 month						
Yes	42	1.0 (0.4–2.5)	12	0.2 (< 0.1–0.5)	54	0.6 (0.3–1.4)*
No	2621	99.1 (97.6–99.6)	1523	99.8 (99.5–99.9)	4144	99.4 (98.6–99.8)
Travel to another country ever						
Yes	566	19.9 (15.6–25.1)	356	18.6 (15.3–22.3)	922	19.3 (15.7–23.4)
No	2098	80.1 (74.9–84.4)	1181	81.4 (77.7–84.7)	3279	80.8 (76.6–84.3)

* p < 0.05 for the difference between round 1 and round 2

### Insecticide-treated net (ITN) use

In the first round only 33% of workers reported habitually using an ITN as a method of malaria prevention. This increased substantially to 56% in round 2 (p < 0.001). Of note PSK distributed long-lasting ITNs between the two survey rounds and in the 2nd round survey over 80% of people reported having received a net, the majority of which were reported to be ITNs received within the previous 2 months. However less than 60% of the temporary workers reported having received a net compared to 90% of permanent staff (p < 0.001).

### Forest exposure and mobility

Although one fifth of workers reported having visited the forest in the previous 1 month, less than 2% had stayed overnight. About one fifth had travelled outside the commune within the previous 1 month of the survey with significantly more (35%) in the 2nd round than the 1st round (9%) (p < 0.001). This is most probably because the 2nd round was conducted just after the Pchum Ben holidays when most Cambodians try to travel back to their home villages. About one fifth of workers had travelled abroad, but less than 1% had done so in the previous 1 month.

### Malaria prevalence

Overall malaria prevalence by PCR was low with little difference between the rounds. In the 1st round 39 plantation workers tested positive (adjusted prevalence rate of 0.9%), which included 25 *Plasmodium falciparum* and 14 *Plasmodium vivax* infections, giving adjusted prevalence rates of 0.6% and 0.3% respectively. In the 2nd round, PCR positivity was 37 (adjusted prevalence of 1.1%), which included 20 *P. falciparum*, 16 *P. vivax* and 1 mixed infection, giving adjusted prevalence rates of 0.6% and 0.5% and 0.01%, respectively (Fig. 3). Prevalence was only 0.2% in family members in both seasons. Only one sample, had a sufficiently high parasite density for the K13 marker to be sequenced, and tested negative for mutations.

By RDT among plantation workers, there were four positive tests in the first round (one for *P. falciparum* and three for *P. vivax*) and in the second round 16 individuals tested positive (seven for *P. falciparum* and nine for *P. vivax*). No family members tested positive by RDT in the first round while two tested positive in round two (one for *P. falciparum* and one for *P. vivax*). Further analysis below is based on PCR results.

**Fig. 4:**
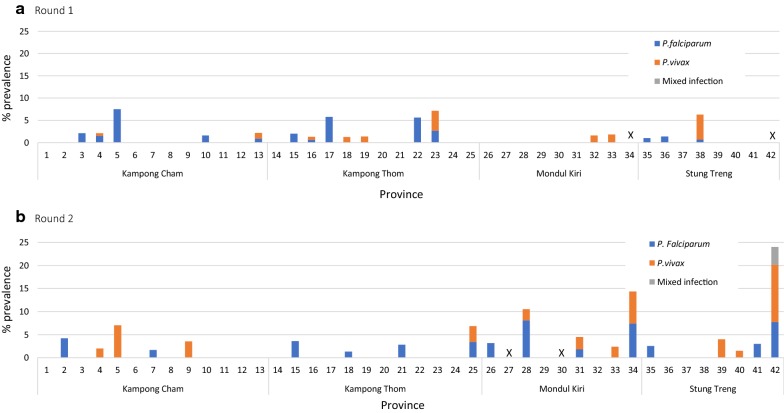
Prevalence of malaria among plantation workers by species by round. X denotes plantations that were not visited during that round of data collection

There was marked heterogeneity in PCR prevalence by plantation as shown in Fig. 4. In both rounds, no malaria was found in just over half of the plantations and *P. falciparum* was only found in 13 out of the 40 plantations visited in each round. However the plantations in which malaria was found, the type of malaria and ranking in terms of prevalence changed between rounds with only 11 plantations having 0% prevalence in both rounds.

In the first round, there was a significantly higher prevalence of 1.6% among small plantations (below 6200 ha) than among large plantation (above 6200 ha), which had a prevalence of 0.4% (p = 0.03), leading to a change in sampling design for the second round of data collection. A higher prevalence was also found in the second round (1.5% on small plantations and 0.9% on larger plantations), but this was not significant.

In the 2nd round, one plantation had a prevalence of 24% (8% *P. falciparum*, 12% *P. vivax* and 4% mixed infection), and another had prevalence of 13.3%, but neither of these plantations had been included in the 1st round. The plantation with a prevalence of 24% was relatively small (500 ha), with only 27 workers and was located within the forest, by a river, and was long in shape. All of the PCR positive individuals were permanent plantation workers or their family members. The small size of this plantation, and its elongated shape meant that the workers were in close proximity to the forest. There was no obvious pattern among the parasite positive workers in terms of individual characteristics.

### Risk factors for parasitaemia

Risk factors associated with malaria prevalence were analysed using bivariate and multivariate analysis using combined data from rounds 1 and 2 (Table 4). In the bivariate analysis, four factors were found to be significant at p ≤ 0.05: age under 30 years; male; lack of reported habitual use of a treated net; and having travelled to another commune in the previous month. In the multivariate analysis, these four risk factors all remained significant.

**Table 4:** Malaria PCR prevalence among plantation workers (adjusted for survey design) and odds of being infected, by risk factor

Risk factor	N	Number PCR positive for malaria	% Adjusted PCR prevalence for malaria (95% CIs)	Unadjusted OR	p-value	Adjusted OR	p-value
Round							
Round 1 (June 2014)	2699	39	0.9 (0.4–1.7)	1	0.60	1	0.71
Round 2 (October 2014)	1536	37	1.1 (0.6–2.1)	1.3 (0.5–3.3)		1.2 (0.4–3.7)	
Gender							
Male	2431	51	1.3 (0.8–2.1)	1	0.01	1	0.03
Female	1768	25	0.5 (0.3–1.1)	0.4 (0.2–0.8)		0.4 (0.2–0.9)	
Age group (years)							
15–30	2208	54	1.6 (1.0–2.4)	1	< 0.01	1	< 0.01
31+	1990	22	0.4 (0.2–0.7)	0.2 (0.1–0.4)		0.2 (0.1–0.4)	
Education							
No or some primary	2925	48	0.9 (0.6–1.6)	1	0.89		
Some Secondary	960	22	1.1 (0.6–2.1)	1.2 (0.6–2.2)			
Completed secondary or higher	313	6	0.8 (0.3–2.2)	0.9 (0.3–2.4)			
Residence status							
Temporary	1682	29	1.2 (0.7–2.1)	1	0.33	1	0.87
Permanent	2516	47	0.9 (0.5–1.6)	0.7 (0.3–1.5)		0.9 (0.5–1.8)	
Type of house							
House	2294	48	1.3 (0.8–2.0)	1	0.52		
Barrack	1488	17	0.6 (0.2–1.4)	0.4 (0.1–1.2)			
Tent or temporary structure	415	11	1.7 (0.7–4.4)	1.4 (0.5–3.9)			
Reported habitual use of treated net as a malaria prevention method at night							
Yes	1702	25	0.5 (0.3–0.9)	1	< 0.01	1	0.01
No	2497	51	1.4 (0.8–2.2)	2.8 (1.5–5.6)		2.9 (1.3–6.4)	
Use of treated net the previous night							
Yes	1898	28	0.6 (0.3–1.2)	1	0.10		
No	2300	48	1.3 (0.7–2.4)	2.2 (0.9–5.6)			
Forest exposure in the previous month							
Yes	1117	18	0.9 (0.4–2.0)	1	0.91	1	0.43
No	3082	58	1.0 (0.6–1.6)	1.0 (0.5–2.3)		1.4 (0.6–3.4)	
Overnight forest exposure in last 1 month							
Yes	99	1	2.2 (0.4–12.1)	1	0.33		
No	4100	75	0.9 (0.6–1.5)	0.4 (0.1–2.5)			
Main Daytime work							
Tapping rubber	1114	18	1.0 (0.5–2.1)	1	0.97		
Planting/caring for young plants	2082	40	1.0 (0.5–1.8)	1.0 (0.4–2.7)			
Clearing forest	304	3	0.4 (0.1–1.4)	0.4 (0.1–1.8)			
Other	699	14	1.1 (0.4–2.5)	1.0 (0.4–3.0)			
Main Night time work							
Tapping rubber	948	19	1.1 (0.5–2.4)	1	0.48		
Does not work	3031	54	0.9 (0.5–1.6)	0.8 (0.3–2.0)			
Other	219	3	0.5 (0.1–2.1)	0.5 (0.1–2.1)			
Travel outside of the commune in the previous 1 month							
Yes	793	20	1.7 (0.9–3.5)	1	0.05	1	0.01
No	3406	56	0.8 (0.4–1.3)	0.4 (0.2–1.0)		0.4 (0.2–0.8)	
Plantation size square root increase			–	0.9 (0.7–1.1)	0.34	0.9 (0.7–1.2)	0.61
Age of plantation in years				0.98 (0.85–1.14)	0.89		
Forest cover in surrounding 5 km buffer zone of plantation				1.7 (0.9–3.3)	0.12	1.6 (0.8–3.2)	0.14

Male plantation workers had 2.4 times the odds of being infected as female workers (p = 0.03). Workers under 30 years had five times the odds of being infected than those over 30 (p < 0.01). Workers who did not regularly use a treated net had 2.9 times the odds of infection (p = 0.01) and people who had travelled had 2.5 times the odds of being infected (p = 0.01).

Risk factors appeared to differ by round (Additional file 1). In round one, only age was found to contribute to the likelihood of being infected with malaria whereas in round 2 the other risk factors mentioned above became significant, in particular forest cover surrounding the plantation being found to affect the likelihood of infection, with a 20% increase in surrounding forest cover being associated with an 2.6 increase in likelihood (1.2–5.5) (p < 0.01).

### Risk factors by species

When broken down by species, plantation size was a significant risk factor for *P. falciparum* infection (Additional file 2). As the square root of the plantation size decreased by 10, the likelihood of infection increased by 1.3, meaning that workers on smaller plantations had a higher risk of infection than those on bigger plantations (p < 0.01). Age, reported treated net use and recent travel also remained significant risk factors for *P. falciparum* infection in the multivariate analysis.

Age, gender and reported net usage were also found to be risk factors for infection by *P. vivax* (Additional file 3). However, plantation size was not found to play an important role, although the data suggests that plantation proximity to forest was associated with malaria infection, with workers from plantations closer to forest cover being 2.1 times as likely to have *P. vivax* infection (p = 0.09).

### Mobility patterns

**Fig. 5:**
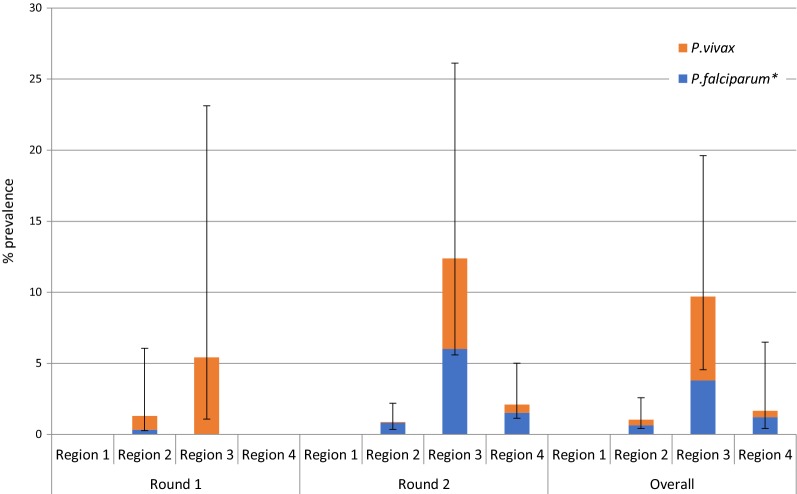
Malaria prevalence among plantation workers by species by region of the country visited in the previous 1 month. Region 1: North-west: Banteay Meanchay, Battambang, Koh Kong, Pursat, Siem Reap, Oddar[a1] Meanchay and Siem Reap. Region 2: Central: Kampong Cham, Kampong Thom, Kampong Chhnang, Kampot, Kampong Speu and Preah Vihear. Region 3: North-east: Mondul Kiri, Kratie, Stung Treng and Ratana Kiri. Region 4: South: Kandal, Phnom Penh, Prey Veng, Sihanoukville, Takeo, Svay Rieng and Kep. Asterisk: There were no mixed infections among people who had travelled in the previous 1 month

Destination of recent travel was an important risk factor for parasitaemia (Fig. 5). Overall ten percent of the individuals who had travelled to the North-eastern provinces of Mondul Kiri, Kratie, Stung Treng or Ratana Kiri within the previous 1 month were PCR positive for malaria, compared with 0–3% prevalence among people who had travelled to other regions (p < 0.01 overall and in round two). None of the those who had travelled to another country in the previous month were infected with malaria.

## Discussion

This study was designed to assess the prevalence of malaria infection in rubber plantation workers in Cambodia, and to describe the risk factors in order to inform the design of subsequent malaria interventions.

There were several important findings. Overall malaria prevalence was lower than expected (around 1% by PCR in workers, and < 0.2% in family members,) and was very heterogenous, with smaller plantations and those that were surrounded by more forest tending towards have a higher prevalence. Unexpectedly, overall prevalence did not differ significantly between the two rounds, nor between temporary (“mobile and migrant”) workers compared to their more settled counterparts. One of the highest risk factors for malaria infection was reported recent travel to the Northeastern provinces. These and other findings and their implications are discussed in more detail below.

Previous studies have reported that people working in rubber plantations are at greater risk of getting malaria [11], with one study in Thailand reporting that daily workers who work on rubber plantations were 2.9 times more likely to be affected by malaria than other villagers [26]. In this survey, there was a low prevalence of malaria overall among rubber plantation workers with substantial proportion of infections being due to *P. vivax*. It is not possible to differentiate between novel and relapsing infections. Similar to village based data, the survey showed that malaria in plantations was highly heterogenous [33]. However, the distribution across the plantations changed between the two rounds. These findings may be due to a number of factors. In South East Asia the available entomological evidence suggests that the favoured environments for the predominant malaria vectors is in dense primary forest [24, 34], while the risk for people who live in nearby villages without going to forested areas remains low [35]. Therefore, one would expect that malaria risk would vary with the proximity of workers to surrounding primary forest and travel into it. In this study, there was some evidence that the more forest there was surrounding a plantation, the higher the risk, as found in the second round of data collection. Indeed the plantation with the highest malaria prevalence (> 24%) was a small elongated plantation surrounded by forest.

Workers involved in clearing primary forest in preparation for planting, and those who venture into the forest for non-plantation related work, for example hunting, logging, and collecting forest products, would also be expected to be at high risk. In this study, most of the plantations were quite established (median age of 7 years) and the majority of workers were engaged in rubber tapping, with very few engaged in clearing forest, therefore the sample size may have been too small to show an association between age of plantation or the main type of work with risk of infection. However, travel outside the commune was associated with highest risk (adjusted OR of 2.5) in multivariable analysis, with travel to the Northeastern provinces, where many workers travel to engage in logging and other forest work, having the highest risk of malaria. This suggests that on mature plantations, plantation work itself (i.e. rubber tapping) may not be the major risk factor for malaria in this population.

There was little difference between the two survey rounds which had originally been intended to measure prevalence during the dry, low transmission season and the wet, higher transmission season. The lack of difference may have been due to a number of reasons. Firstly, due to unavoidable delays the first round was carried out in June, during the transition from dry to wet season, therefore, malaria transmission may have already been on the increase. Secondly, ITNs were distributed between the two rounds and this was associated with a significant increase in their reported use from 33 to 56%, and would be expected to decrease the rate of infection. Indeed workers who reported regularly using an ITN were nearly three times less likely to have malaria, suggesting that despite reservations about their utility in this setting, they have an important role. Of note, the two plantations that were surveyed in the 2nd round that were included as replacements, were also the two with the highest prevalence. Had there been no change in the plantations between rounds, it could be expected that the prevalence would have been even lower in the 2nd round.

It was also expected that a higher proportion of the plantation workers would be temporary mobile and migrant workers and that they would have a higher risk of being malaria infected than permanent residents. Other studies have found that MMPs are at higher risk of malaria than permanent more settled communities [36]. However, the majority of workers were permanent residents and there was no significant difference in malaria risk between the two groups. This was despite permanent residents being over 30% more likely to have received an ITN than the temporary workers. The latter however was a useful finding in illustrating a discrepancy in who receives services provided on site with implications which should be addressed in follow-on net distributions to ensure equity of access and protection for those workers who are more mobile. A continual distribution of nets to new workers at the commencement of work might result in more equitable net usage than one-off distributions.

Interventions that have been discussed in the context of malaria elimination include mass drug administration and mass or focal screening and treatment, however given the heterogeneity of malaria risk between plantations, it would be important to target such interventions appropriately. One of the highest risk factors for malaria was recent travel to the Northeastern provinces, presumably in order to work in the forest. Village-based pro-active case screening of individuals who have recently returned from the forest has been shown to be successful at identifying asymptomatic infected individuals in Cambodia [37], so expanding the existing PMW programme from the passive case detection to include active case detection could lead to identification of more asymptomatic infections. Screening and treating family and households around positive cases may not be an efficient method to capture cases due to the low positivity rate found among family members. As the majority of individuals testing positive by PCR were RDT negative and asymptomatic, relying on conventional RDTs would miss the majority of infected workers. A recent study utilising highly sensitive RDTs under operational conditions in Cambodia suggests they do not pick up more infections than conventional RDTs in this setting [38]. Finally, the strong association between net usage and infection indicates the continued importance of ensuring all plantation workers are using nets.

### Study limitations

The following study limitations are identified. First, plantations visited during this study had a working relationship with PSK, however gaining entry remained challenging, and two plantations had to be replaced in the second round for this reason. Second, due to the quasi-legal nature of work on some sites, land concession disputes and issues related to labour regulations, the management on five sites only permitted the survey teams access to certain subgroups of workers, which meant that the sampling method according to the study protocol was not possible. Third, the most transient workers (and, therefore, potentially the most at risk) employed on an ad hoc basis may have been missed from the census worker lists resulting in an underestimate of malaria prevalence. Other approaches such as ethnographic studies might be necessary to get a better understanding of this population. The data reported by the workers may have been subject to recall bias. Finally, there were challenges in obtaining data on plantations and defining their characteristics. There is no universally accepted definition of “forest” on which to base the “forest proximity” variable. Additionally, five plantations were not accounted for on the available maps and therefore forest proximity could not be calculated. The reported size of the plantations was based on economic land concession data, which did not necessarily represent the actual area under plantation, and may have reduced the relationship between size of plantation and malaria prevalence. Finally, defining the age of plantation was sometimes challenging as they sometimes underwent changes in management or different stages of clearing and planting and the age reported did not always reflect the same thing.

## Conclusions

With Cambodia’s *P. falciparum* elimination target on the horizon, identifying every single malaria case will become increasingly important. Real time surveillance at every point where fever cases present are critical. Plantation workers are relatively accessible compared to some other at-risk groups and will likely remain a high priority. Expanding the PMW programme, possibly by including active-case detection, and continuous surveillance of plantation level data should enable swift action to be taken, potentially focusing on the smaller plantations and those in close proximity to primary forest. Finally, as widely documented elsewhere, as malaria transmission falls, *P. vivax* will become increasingly dominant and without implementing radical cure it is difficult to see how the ambitious targets for malaria elimination will be reached.

## Supplementary information

**Additional file 1.** Malaria prevalence by PCR among plantation workers (adjusted for survey design) and odds of being infected, by risk factor, by round.

**Additional file 2.** *P. falciparum* prevalence by PCR among plantation workers (adjusted for survey design) and odds of being infected, by risk factor.

**Additional file 3.** *P. vivax* prevalence by PCR among plantation workers (adjusted for survey design) and odds of being infected, by risk factor.

## Data Availability

The datasets used and analysed during the current study are available from the corresponding author on reasonable request.

## Abbreviations

CNM: Cambodian National Centre for Parasitology, Entomology and Malaria Control
ITN: Insecticide-treated Net
MMP: Mobile and Migrant Population
PCR: Polymerase Chain Reaction
PMW: Plantation Malaria Worker
PSI: Population Services International
PSK: Population Services Khmer
RDT: Rapid Diagnostic Test
VMW: Village Malaria Worker
