# Supplementary material for: Rubber plantations and drug resistant malaria: a cross-sectional survey in Cambodia
Source: Malar J. 2019 Nov 27;18:379. doi: 10.1186/s12936-019-3000-y (PMC6882203; doi:10.1186/s12936-019-3000-y)
Supplement: Supplementary file 1 — Additional file 1. Malaria prevalence by PCR among plantation workers (adjusted for survey design) and odds of being infected, by risk factor, by round. [file 12936_2019_3000_MOESM1_ESM.docx]

Additional files:

**Additional file 1:** Malaria PCR prevalence among plantation workers (adjusted for survey design) and odds of being infected by risk factor, by round

1. **Round 1**

| Risk factor | N | Number of positive cases | Prevalence | Unadjusted OR | p-value | Adjusted OR |  |
| --- | --- | --- | --- | --- | --- | --- | --- |
| **Gender**  Male  Female | 1,493  1,171 | 26  13 | 1.0 (0.5-2.2)  0.6 (0.3-1.4) | 1  0.6 (0.3-.1.3) | 0.17 | 1  0.7 (0.3-1.6) | 0.32 |
| **Age group**  15-30  31+ | 1,405  1,259 | 27  12 | 1.3 (0.7-2.6)  0.4 (0.2-0.9) | 1  0.3 (0.1-0.6) | <0.01 | 1  0.3 (0.1-0.7) | <0.01 |
| **Education**  No or some primary  Some Secondary  Completed secondary or higher | 1,839  613  211 | 22  13  4 | 0.7 (0.3-1.6)  1.3 (0.6-2.8)  1.0 (0.3-3.4) | 1  1.8 (0.8-4.3)  1.5 (0.4-5.4) | 0.22 |  |  |
| **Residence status**  Temporary  Permanent | 1,207  1,457 | 17  22 | 0.8 (0.4-1.7)  0.9 (0.3-2.2) | 1  1.0 (0.3-3.1) | 0.96 | 1  1.2 (0.4-3.6) | 0.80 |
| **Reported habitual use of treated net as a malaria prevention method at night** Yes  No | 795  1,869 | 7  32 | 0.6 (0.2-1.6)  1.0 (0.5-2.1) | 1  1.7 (0.7-4.3) | 0.25 | 1  1.6 (0.6-4.1) | 0.37 |
| **Use of treated net the previous night**  Yes  No | 927  1,736 | 9  30 | 0.4 (0.2-1.1)  1.1 (0.5-2.4) | 1  2.6 (0.8-8.7) | 0.12 |  |  |
| **Forest exposure in the last one month**  Yes  No | 747  1,917 | 11  28 | 0.7 (0.2-1.9)  0.9 (0.4-2.0) | 1  1.3 (0.4-4.9) | 0.68 | 1  1.2 (0.2-6.3) | 0.82 |
| **Overnight forest exposure in last one month**  Yes  No | 25  2,639 | 0  39 | 0.0  0.9 (0.4-1.7) | 1  - | - |  |  |
| **Daytime work**  Tapping rubber  Planting/ caring for young plants  Clearing forest  Other | 743  1,142  292  487 | 12  13  3  11 | 1.0 (0.3-3.1)  0.6 (0.2-1.3)  0.5 (0.1-1.5)  1.5 (0.6-4.0) | 1  0.6 (0.1-2.4)  0.5 (0.1-2.4)  1.7 (0.4-7.1) | 0.72 |  |  |
| **Nighttime work**  Tapping rubber  Does not work  Other | 657  1,811  156 | 13  23  3 | 1.0 (0.3-3.3)  0.7 (0.4-1.4)  1.0 (0.2-3.8) | 1  0.7 (0.2-2.6)  0.9 (0.2-5.3) | 0.69 |  |  |
| **Travelled outside of the commune in the previous one month**  Yes  No | 211  2,454 | 2  37 | 0.8 (0.1-4.4)  0.9 (0.4-1.8) | 1  1.1 (0.2-7.4) | 0.91 | 1  1.1 (0.2-9.2) | 0.94 |
| **Plantation size square root increase** |  |  |  | 0.9 (0.8-1.0) | 0.05 | 0.9 (0.7-1.0) | 0.13 |
| **Age of plantation in years** |  |  |  | 1.0 (0.9-1.2) | 0.45 |  |  |
| **Forest cover in surrounding 5km buffer zone of plantation** |  |  |  | 1.1 (0.4-2.6) | 0.91 | 0.8 (0.3-2.6) | 0.73 |

1. **Round 2**

| Risk factor | N | Number of positive | Prevalence | Unadjusted OR | p-value | Adjusted OR | p-value |
| --- | --- | --- | --- | --- | --- | --- | --- |
| **Gender**  Male  Female | 938  597 | 25  12 | 1.5 (0.8-3.1)  0.5 (0.2-1.2) | 1  0.3 (0.1-0.8) | 0.02 | 1  0.3 (0.1-1.0) | 0.05 |
| **Age group**  15-30  31+ | 803  731 | 27  10 | 1.8 (0.9-3.4)  0.3 (0.1-0.9) | 1  0.2 (0.1-0.5) | <0.01 | 1  0.2 (0.1-0.6) | <0.01 |
| **Education**  No or some primary  Some Secondary  Completed secondary or higher | 1,086  347  102 | 26  9  2 | 1.2 (0.6-2.4)  1.0 (0.3-2.6)  0.6 (0.1-2.7) | 1  0.8 (0.3-2.2)  0.4 (0.1-2.5) | 0.40 |  |  |
| **Residence status**  Temporary  Permanent | 475  1,059 | 12  25 | 1.9 (0.9-4.3)  0.9 (0.4-1.9) | 1  0.4 (1.6-1.2) | 0.12 | 1  0.7 (0.3-1.8) | 0.46 |
| **Reported habitual use of treated net as a malaria prevention method at night**  Yes  No | 907  628 | 18  19 | 0.4 (0.2-0.9)  1.9 (0.9-4.0) | 1  4.7 (1.8-12.3) | <0.01 | 1  5.1 (1.9-13.8) | <0.01 |
| **Use of treated net the previous night**  Yes  No | 971  565 | 19  18 | 0.7 (0.3-1.8)  1.7 (0.7-4.0) | 1  2.4 (0.7-8.6) | 0.17 |  |  |
| **Forest exposure in the last one month**  Yes  No | 370  1,165 | 7  30 | 1.3 (0.5-3.1)  1.1 (0.5-2.1) | 1  0.8 (0.3-2.0) | 0.69 | 1  1.6 (0.5-5.2) | 0.42 |
| **Overnight forest exposure in last one month**  Yes  No | 74  1,461 | 1  36 | 2.9 (0.5-14.8)  1.1 (0.6-2.0) | 1  0.3 (0.1-2.0) | 0.24 |  |  |
| **Daytime work**  Tapping rubber  Planting/ caring for young plants  Clearing forest  Other | 371  940  12  207 | 6  27  0  3 | 1. (0.3-3.1)   1.3 (0.7-2.7)  0.0  0.4 (0.0-2.1) | 1  1.3 (0.3-5.4)  -  0.4 (0.1-2.6) | 0.64 |  |  |
| **Nighttime work**  Tapping rubber  Does not work  Other | 291  1,180  63 | 6  31  0 | 1.2 (0.4-4.1)  1.1 (0.6-2.2)  0.0 | 1  0.9 (0.2-3.6)  - | 0.59 |  |  |
| **Travelled outside of the communie in the previous one month**  Yes  No | 582  954 | 18  19 | 1. (0.9-4.3)   0.6 (0.2-1.5) | 1  0.3 (0.1-0.8) | 0.02 | 1  0.2 (0.1-0.8) | 0.02 |
| **Plantation size square root increase** |  |  | - | 0.9 (0.701.2) | 0.55 | 0.9 (0.7-1.3) | 0.71 |
| **Age of plantation in years** |  |  |  | 0.9 (0.7-1.3) | 0.55 |  |  |
| **Forest cover in surrounding 5km buffer zone of plantation** |  |  |  | 1.1 (0.4-2.6) | 0.88 | 2.6 (1.2-5.5) | 0.01 |
