# Supplementary material for: Rubber plantations and drug resistant malaria: a cross-sectional survey in Cambodia
Source: Malar J. 2019 Nov 27;18:379. doi: 10.1186/s12936-019-3000-y (PMC6882203; doi:10.1186/s12936-019-3000-y)
Supplement: Supplementary file 2 — Additional file 2. P. falciparum prevalence by PCR among plantation workers (adjusted for survey design) and odds of being infected, by risk factor. [file 12936_2019_3000_MOESM2_ESM.docx]

**Additional file 2:** *P. falciparum* prevalence (adjusted for survey design) by risk factor, and odds of being infected with malaria by risk factor

| Risk factor |  |  | Prevalence | Unadjusted OR | p-value | Adjusted OR |  |
| --- | --- | --- | --- | --- | --- | --- | --- |
| **Round** |  |  |  |  |  |  |  |
| Round 1 (June 2014) | 2,699 | 25 | 0.6(0.3-1.2) | 1 | 0.90 | 1 | 0.75 |
| Round 2 (October 2014) | 1,536 | 21 | 0.6(0.3-1.3) | 1.1(0.4-3.3) |  | 0.8 (0.3-2.6) |  |
| **Gender** |  |  |  |  |  |  |  |
| Male | 2,431 | 30 | 0.7 (0.4-1.3) | 1 | 0.19 | 1 | 0.32 |
| Female | 1,768 | 16 | 0.4 (0.2-0.9) | 0.6 (0.3-1.3) |  | 0.6 (0.3-1.6) |  |
| **Age group** |  |  |  |  |  |  |  |
| 15-30 | 2,208 | 31 | 0.9 (0.5-1.6) | 1 | 0.04 | 1 | 0.01 |
| 31+ | 1,990 | 15 | 0.2 (0.1-0.5) | 0.3 (0.1-0.6) |  | 0.3 (0.1-0.7) |  |
| **Education** |  |  |  |  |  |  |  |
| No or some primary | 2,925 | 28 | 0.6 (0.3-1.1) | 1 | 0.82 |  |  |
| Some Secondary | 960 | 13 | 0.6 (0.3-1.3) | 1.1 (0.4-2.6) |  |  |  |
| Completed secondary or higher | 313 | 5 | 0.6 (0.2-1.9) | 1.1 (0.3-3.9) |  |  |  |
| **Residence status** |  |  |  |  |  |  |  |
| Temporary | 1,682 | 18 | 0.7 (0.3-1.4) | 1 | 0.67 | 1 | 0.58 |
| Permanent | 2,516 | 28 | 0.5 (0.3-1.1) | 0.8 (0.3-2.1) |  | 1.3 (0.5-3.3) |  |
| **Type of house**  House  Barrack  Tent or temporary structure | 2,294  1,488  415 | 24  15  7 | 0.7 (0.4-1.3)  0.4 (0.2-1.2)  0.8 (0.2-3.1) | 1  0.6 (0.2-2.0)  1.2 (0.3-5.3) | 0.71 |  |  |
| **Reported habitual use of treated net as a malaria prevention method at night** |  |  |  |  |  |  |  |
| Yes | 1,702 | 16 | 0.3 (0.2-0.7) | 1 | 0.05 | 1 | 0.05 |
| No | 2,497 | 30 | 0.8 (0.4-1.4) | 2.3 (1.0-5.3) |  | 2.3 (0.8-6.8) |  |
| **Reported use of treated net the previous night**  Yes  No | 1,898  2,300 | 18  28 | 0.5 (0.2-1.1)  0.7 (0.3-1.4) | 1  1.5 (0.5-4.9) | 0.50 |  |  |
| **Forest exposure in the last one month** |  |  |  |  |  |  |  |
| Yes | 1,117 | 11 | 0.5 (0.2-1.3) | 1 | 0.82 | 1 | 0.50 |
| No | 3,082 | 35 | 0.6 (0.3-1.1) | 1.1 (0.4-3.1) |  | 1.5 (0.5-4.9) |  |
| **Overnight forest exposure in last one month** |  |  |  |  |  |  |  |
| Yes | 99 | 0 | 0 | 1 | - |  |  |
| No | 4,100 | 46 | 0.6 (0.3-1.2) | - |  |  |  |
| **Main daytime work** |  |  |  |  |  |  |  |
| Tapping rubber | 1,114 | 13 | 0.7 (0.3-1.7) | 1 | 0.32 |  |  |
| Planting/ caring for young plants | 2,082 | 23 | 0.5 (0.2-1.0) | 0.7 (0.2-2.1) |  |  |  |
| Clearing forest | 304 | 1 | 0.2 (<0.1-1.2) | 0.2 (0.0-2.0) |  |  |  |
| Other | 699 | 8 | 0.4 (0.1-1.1) | 0.5 (0.2-1.8) |  |  |  |
| **Main nighttime work** |  |  |  |  |  |  |  |
| Tapping rubber | 948 | 13 | 0.8 (0.3-2.0) | 1 | 0.21 |  |  |
| Does not work | 3,031 | 31 | 0.4 (0.2-0.8) | 0.5(0.2-1.5) |  |  |  |
| Other | 219 | 2 | 0.3 (<0.01-1.8) | 0.4 (0.1-2.6) |  |  |  |
| **Travelled outside of the commune in the previous one month** |  |  |  |  |  |  |  |
| Yes | 793 | 12 | 1.2 (0.5-2.8) | 1 | 0.07 | 1 | <0.01 |
| No | 3,406 | 34 | 0.4 (0.2-0.8) | 0.4 (0.1-1.1) |  | 0.2 (0.1-0.6) |  |
| **Plantation size square root increase** |  |  |  | 0.8 (0.7-0.9) | <0.01 | 0.8 (0.7-0.9) | <0.01 |
| **Age of plantation in years** |  |  |  | 1.0 (0.9-1.2) | 0.87 |  |  |
| **Forest cover in surrounding 5km buffer zone of plantation** |  |  |  | 1.6 (0.7-3.6) | 0.22 | 1.4 (0.6-3.5) | 0.48 |
|  |  |  |  |  |  |  |  |
