# Supplementary material for: Rubber plantations and drug resistant malaria: a cross-sectional survey in Cambodia
Source: Malar J. 2019 Nov 27;18:379. doi: 10.1186/s12936-019-3000-y (PMC6882203; doi:10.1186/s12936-019-3000-y)
Supplement: Supplementary file 3 — Additional file 3. P. vivax prevalence by PCR among plantation workers (adjusted for survey design) and odds of being infected, by risk factor. [file 12936_2019_3000_MOESM3_ESM.docx]

**Additional file 3:** *P. vivax* prevalence among plantation workers (adjusted for survey design) by risk factor, and odds of being infected with malaria by risk factor*anne*

| Risk factor |  | Number of parasite positive | Prevalence | Unadjusted OR | p-value | Adjusted OR |  |
| --- | --- | --- | --- | --- | --- | --- | --- |
| **Round** |  |  |  |  |  |  |  |
| Round 1 (June 2014) | 2,699 | 14 | 0.3(0.1-0.7) | 1 | 0.36 | 1 | 0.36 |
| Round 2 (October 2014) | 1,536 | 17 | 0.5(0.2-1.3) | 1.7 (0.5-5.5) |  | 1.9 (0.5-8.0) |  |
| **Gender** |  |  |  |  |  |  |  |
| Male | 2,431 | 22 | 0.6 (0.3-1.2) | 1 | <0.01 | 1 | 0.01 |
| Female | 1,768 | 9 | 0.1 (<0.0.1-0.3) | 0.2 (0.1-0.6) |  | 0.2 (0.1-0.6) |  |
| **Age group** |  |  |  |  |  |  |  |
| 15-30 | 2,208 | 24 | 0.7 (0.3-1.3) | 1 | <0.01 | 1 | 0.01 |
| 31+ | 1,990 | 7 | 0.1 (<0.1-0.4) | 0.2(0.1-0.6) |  | 0.2 (0.1-0.6) |  |
| **Education** |  |  |  |  |  |  |  |
| No or some primary | 2,925 | 21 | 0.4(0.2-0.8) | 1 | 0.88 |  |  |
| Some Secondary | 960 | 9 | 0.5(0.2-1.4) | 1.3(0.5-3.4) |  |  |  |
| Completed secondary or higher | 313 | 1 | 0.2(<0.01-1.2) | 0.4(0.1-2.9) |  |  |  |
| **Residence status** |  |  |  |  |  |  |  |
| Temporary | 1,682 | 11 | 0.6 (0.3-1.2) | 1 | 0.22 | 1 | 0.56 |
| Permanent | 2,516 | 20 | 0.3 (0.1-0.7) | 0.6 (0.2-1.4) |  | 0.8 (0.3-1.9) |  |
| **Type of house**  House  Barrack  Tent or temporary structure | 2,294  1,488  415 | 24  2  5 | 0.6 (0.3-1.0)  0.1 (0.0-0.6)  1.0 (0.3-3.4) | 1  0.2 (0.1-1.1)  1.7 (0.5-6.6) | 0.62 |  |  |
| **Reported habitual use of treated net as a malaria prevention method at night** |  |  |  |  |  |  |  |
| Yes | 1,702 | 10 | 0.2 (<0.01-0.4) | 1 | 0.01 | 1 | 0.02 |
| No | 2,497 | 21 | 0.6 (0.3-1.2) | 4.0 (1.4-11.4) |  | 3.7 (1.2-11.7) |  |
| **Use of treated net the previous night**  Yes  No | 1,898  2,300 | 11  20 | 0.2 (<0.01-0.3)  0.6 (0.3-1.3) | 1  4.2 (1.6-10.8) | <0.01 |  |  |
| **Forest exposure in the last one month** |  |  |  |  |  |  |  |
| Yes | 1,117 | 7 | 0.4(0.1-1.1) | 1 | 0.94 | 1 | 0.57 |
| No | 3,082 | 24 | 0.4(0.2-0.8) | 1.0 (0.3-3.1) |  | 1.4 (0.4-4.6) |  |
| **Overnight forest exposure in last one month** |  |  |  |  |  |  |  |
| Yes | 99 | 1 | 2.2(0.4-12.1) | 1 | 0.03 | * |  |
| No | 4,100 | 30 | 0.4(0.2-0.7) | 0.2(0.0-0.8) |  |  |  |
| **Main Daytime work** |  |  |  |  |  |  |  |
| Tapping rubber | 1,114 | 5 | 0.3 (<0.01-0.8) | 1 | 0.19 |  |  |
| Planting/ caring for young plants | 2,082 | 18 | 0.5 (0.2-1.2) | 1.9 (0.5-8.4) |  |  |  |
| Clearing forest | 304 | 2 | 0.3 (<0.01-1.1) | 1.0 (0.2-6.3) |  |  |  |
| Other | 699 | 6 | 0.7 (0.2-2.2) | 2.5 (0.6-12.0) |  |  |  |
| **Main Nighttime work** |  |  |  |  |  |  |  |
| Tapping rubber | 948 | 6 | 0.3 (<0.01-0.9) | 1 | 0.61 |  |  |
| Does not work | 3,031 | 24 | 0.5 (0.2-1.1) | 1.6 (0.4-6.2) |  |  |  |
| Other | 219 | 1 | 0.2 (<0.01-1.6) | 0.7 (0.1-6.2) |  |  |  |
| **Travelled outside the commune in the previous one month** |  |  |  |  |  |  |  |
| Yes | 793 | 8 | 0.6(0.2-1.5) | 1 | 0.23 | 1 | 0.52 |
| No | 3,406 | 23 | 0.3(0.2-0.7) | 0.6(0.2-1.4) |  | 0.7 (0.2-2.2) |  |
| **Plantation size square root increase** |  |  |  | 1.0 (0.7-1.4) | 0.88 | 1.1 (0.8-1.4) | 0.63 |
| **Age of plantation in years** |  |  |  | 0.9 (0.6-1.4) | 0.71 |  |  |
| **Forest cover in surrounding 5km buffer zone of plantation** |  |  |  | 1.8 (0.8-3.8) | 0.15 | 2.1 (0.9-4.8) | 0.09 |

***Overnight forest exposure not included in the model despite being significant at p<0.05, due to the low number of people who stay overnight in the forest, which makes the variable not suitable for the model
